# Kynurenine pathway dysregulation via loss of QPRT drives declines in activity and altered metabolism in mice

**DOI:** 10.1007/s11357-025-01735-1

**Published:** 2025-07-10

**Authors:** Reyhan Westbrook, Vinal Menon, Joy Cagmat, Timothy Garrett, Robert Schwarcz, Jeremy Walston, Peter M. Abadir, Tae Chung

**Affiliations:** 1 Division of Geriatric Medicine and Gerontology, Johns Hopkins University School of Medicine, Baltimore, MD USA; 2 Department of Physical Medicine and Rehabilitation, Johns Hopkins University School of Medicine, Baltimore, MD USA; 3 Southeast Center for Integrated Metabolomics, University of Florida, Gainesville, FL USA; 4 Department of Psychiatry, Maryland Psychiatric Research Center, University of Maryland School of Medicine, Baltimore, MD USA; 5 Department of Neurology, Johns Hopkins University School of Medicine, Baltimore, MD USA

**Keywords:** Quinolinic acid, Kynurenine, Nicotinamide, Mice, Indirect calorimetry, Metabolism

## Abstract

Chronic inflammatory pathway activation increases with age and is epidemiologically linked to multiple aging-related pathophysiological processes, phenotypes such as physical frailty and sarcopenia and early healthspan declines in aging organisms. Despite this, molecular mechanisms that directly connect chronic inflammation to these conditions remain poorly characterized. We hypothesize that chronic inflammation contributes to the development of age-related phenotypes by increasing the degradation of dietary tryptophan into multiple metabolites with unique physiological properties, called kynurenines, via the ‘kynurenine pathway’ (KP). To understand the impact of elevated KP metabolites on mammalian healthspan we utilized the quinolinate phosphoribosyltransferase knock-out (QPRT^−/−^) mouse which lacks the terminal enzyme of the KP and thus develops increased levels of downstream kynurenines. We tested the effects of this mutation on glucose handling, spontaneous motor activity, body composition and metabolism using indirect calorimetry, in male and female, young, middle aged and older mice. QPRT − / − mice had significantly altered levels of numerous KP metabolites and nicotinamide. Phenotypic characteristics varied in a sex-specific manner with decreased activity, lean mass and VO_2_, and impaired glucose clearance as early as 12 months seen in female QPRT^−/−^ compared to age and sex-matched mice. Male QPRT^−/−^ mice developed reduced lean mass by middle age and had altered respiration and food intake. This data indicates that KP dysregulation can drive declines in activity and alter metabolism in mammals and is a potential target to intervene on frailty and functional decline.

**Supplementary Information:**

The online version contains supplementary material available at 10.1007/s11357-025-01735-1.

## Introduction

Chronic inflammation, as measured by persistently elevated serum levels of inflammatory cytokines, is increasingly recognized as playing an etiological role in the development of multiple adverse conditions related to aging [1–4]. Downstream metabolites of tryptophan called ‘kynurenines’ are modulated by inflammation and may, in turn, influence adverse health outcomes. Kynurenine metabolite imbalance is noted in a number of inflammation-related diseases including cancer [5], metabolic syndrome [6], cardiovascular [7], neurodegenerative [8], and kidney diseases [9] and physical frailty [10]. Together, this data suggests that the KP dysregulation is a common element in the decline of multiple metabolic systems in the context of chronic inflammation-related diseases and syndromes.

The pleiotropic nature of KP links the fluxes of this pathway to diverse aspects of mammalian physiology and suggests important influences on multiple physiological systems. Tryptophan is required for the synthesis of serotonin and melatonin and, through the KP, the synthesis of the energy metabolite and cellular cofactor, nicotinamide adenine dinucleotide (NAD +) [11]. More than 95% of available tryptophan is degraded through the KP, with the conversion of tryptophan to kynurenine, being the rate-limiting step of the pathway [12]. Under basal conditions this step is mediated by liver-specific tryptophan 2,3-dioxygenase (TDO) [12–14]. Extra-hepatic conversion of tryptophan to kynurenine is carried out by indolamine 2,3 dioxygenases (IDO1 & IDO2) [15, 16]. IDO expression is low in most tissues but localized to microenvironments including antigen-presenting immune cells (e.g., dendritic cells, macrophages) [17]. IDO’s expression and activity are dramatically upregulated by inflammatory cytokines (e.g., IFN-γ) and repressed by anti-inflammatory cytokines [18–20]. Kynurenine 3-monooxygenase (KMO) converts kynurenine to 3-hydroxykynurenine, and its expression and activity are also increased by inflammatory cytokines [20–22]. Accordingly, pro-inflammatory signals increase the conversion of tryptophan into kynurenines.

While inflammatory cytokines dramatically induce IDO, which controls the first step in the KP and de novo NAD + synthesis, chronic inflammation [23] and aging [24] are associated with decreased NAD + levels. These seemingly conflicting observations are possibly reconciled by findings from isotope-labeled tryptophan tracer studies on the dynamics of the KP in immune cells [25]. These studies showed that immune-challenged macrophages produced more KP metabolites but NAD + synthesis is suppressed because the conversion of quinolinic acid to NAD +, catalyzed by quinolinate phosphoribosyltransferase (QPRT), becomes limiting. This profile is recapitulated in aging macrophages. This increase in KP intermediates and reduction in NAD +, modulates macrophage and other immune cell behavior during the immune response [25–27]; however, it is not known how chronic elevations in quinolinic acid and reduced NAD + affect metabolism and function in mammals, in vivo.

Chronic inflammation induced KP activation with decreased NAD + synthesis set the premise for our hypothesis that pooling of toxic KP intermediates mediates declines in multiple systems. Quinolinic acid (QA) and other downstream kynurenines are excitotoxins [28], and cause oxidative damage [29–31]. Older mice have exaggerated production [32, 33] and higher serum and tissue levels of inflammatory cytokines and, as a result, have increased levels of kynurenines. Thus, chronic inflammation, regardless of source, sets up this dysregulation in the KP. This chronic inflammation-driven pooling of toxic kynurenines in frailty is confirmed by our own measurements in humans [10].

To pinpoint biological pathways that change with age as well as connect chronic inflammation to functional decline and physical frailty, we used metabolomics to profile downstream KP metabolites in the serum of older adults and found that downstream kynurenines including QA were increased in frail and non-frail subjects compared to young subjects. These metabolites were also significantly correlated with inflammatory cytokine levels reinforcing the connections between toxic kynurenine metabolites and chronic inflammation. We tested whether 3-HK and QA have toxic effects on the murine motor neurons in vitro and saw that toxicity of 3-HK and QA occurred at physiologically relevant levels during inflammation. We hypothesize that it is the chronic exposure to elevations in levels of toxic kynurenines that occurs with chronic inflammation and aging that causes damage to peripheral nerves and other tissues and impacts healthspan.

To test these hypotheses, we have utilized a mouse model with a targeted disruption of the *Qprt* gene locus [34] leading to elevated QA levels systemically, throughout their lifespan (QPRT^−/−^ mouse). We have previously shown that QPRT^−/−^ mice have accelerated neuromuscular decline with aging in a sex-specific pattern [35]. In this study, we have examined the physiological and functional consequences of the loss of *Qprt* expression by measuring kynurenine levels, body composition, glucose handling, indirect calorimetry and ambulatory activity.

## Methods

### Animals

Mutant mice (C57BL/6 J/129S background) were generated by gene trapping using an OmniBank embryonic stem cell line containing an insertion within the first intron of the Qprt gene. A BLAST search of the OmniBank sequence database (Zambrowicz et al., 1998) identified several mouse embryonic stem (ES) cell clones predicted to contain gene trap mutations within the *Qprt* gene (accession NM_133686). ES cell clone OST337946 was selected for further characterization based on the sequence similarity of its 3′ RACE (rapid amplification of cDNA ends) tag with the second exon of the *Qprt* gene. Inverse genomic PCR was used to amplify the vector (EU676804) insertion site from this clone and to localize the insertion mutation to intron 1. Loss of *Qprt* expression was confirmed by reverse transcription (RT)-PCR analysis using primers complementary to exons flanking the insertion. Primers used for genotyping are as follows: knockout/knockout forward (CGACA CCT GGG TTC CGA CTGGT) and reverse (CCTGC CGA GCC TTC AGC ACTGC) 398 bp targeted primers and wild type forward (TGGGC ACA ACA GAC AATCGG) and reverse (ACTTC GCC CAA TAG CAG CCAG) 221 bp primers. Mice were bred and housed at the Johns Hopkins University Bayview Vivarium and maintained under SPF barrier conditions in a 14-h light/10-h dark cycle kept at a temperature automated range between 22 °C ± 3.6 °C (Siemens Building Technologies Inc.). More specifically, animals were housed in high-temperature polycarbonate 75 in2 autoclaved cages in ventilated racks (Allentown Inc.). Bedding consisted of autoclaved corncob from Harlan Teklad. Autoclaved chow (Harlan, Teklad 2018SX) and reverse osmosis–filtered hyperchlorinated water were always provided to animals (Edstrom Industries). Animal cages were changed every 2 weeks within animal research sterile fume hoods (The Baker Co.) disinfected with MB-10 disinfectant spray (Quip Laboratories Inc.). Mice were monitored daily, and body and tissue weights were recorded from each mouse using a standard analytical scale (Mettler Toledo, LCC). This cross-sectional study examined male and female QPRT^−/−^ mice and background matched control mice in three age groups. The young group was 8 months and *n* = 5 per group. The middle aged males were 13.5 months old (QPRT^−/−^ *n* = 12 and controls *n* = 8) and females were aged 13 months (QPRT^−/−^ *n* = 9 and controls, *n* = 11). The old age males were 21 months old (QPRT^−/−^ *n* = 7 and controls *n* = 6) and females were 20 months old (QPRT^−/−^ *n* = 7 and controls *n* = 12).

### GTT

To measure the effect of *Qprt* gene deletion on glucose homeostasis, we performed a glucose tolerance test (GTT). For the glucose tolerance test (GTT), mice were fasted overnight (~ 12 h) and on the subsequent morning, injected i.p. with 2 g glucose/kg body weight. Blood samples (amount to fill capillary in standard glucometer; approximately 0.002 mL per sample) were collected from the tail at baseline, 15, 30, 60 and 120 min after injection. For collection of the first sample, an approximately 3–4 mm knick near the tip of the tail was cut with a sharp razor blade and the tail gently massaged. For collection of consecutive blood sample, the scab was removed with clean tissue.

## Body composition

The amounts of body fluid, fat, and lean body mass were determined in whole live mice. The assessment of total fat, lean, and fluid mass for each individual animal was acquired by nuclear magnetic resonance (NMR) using the Minispec LF90 (Bruker Optics, Billerica, MA). These measurements are then compared to total body mass to acquire the relative percentage of fat, lean and fluid mass.

## Indirect calorimetry and activity

Mouse metabolic rate was assessed by indirect calorimetry in open-circuit oxymax chambers using the Comprehensive Lab Animal Monitoring System (CLAMS; Columbus Instruments, Columbus, OH). Mice (*n* = 10 per age and genotype group) were housed singly with ad libitum access to food and water and maintained at 20–22 °C under a 12:12-h light–dark cycle (light period 0600–1800). Sample air was passed through an oxygen sensor for determination of oxygen content. Oxygen consumption was determined by measuring oxygen concentration in air entering the chamber compared with air leaving the chamber. The sensor was calibrated against a standard gas mix containing defined quantities of oxygen, carbon dioxide and nitrogen. Constant airflow (0.5 L/min) was drawn through the chamber and monitored by a mass sensitive flow meter. The concentrations of oxygen and carbon dioxide were monitored at the inlet and outlet of the sealed chambers to calculate oxygen consumption (VO_2_) and carbon dioxide production (VCO_2_). Other metabolic variables generated include respiratory exchange rate (RER = VCO_2_/VO_2_) energy expenditure (EE = *CV* ∗ *VO*2,*bject*, “calorific value” (CV) = 3.815 + 1.232 ∗ *RER*) Measurement in each chamber was recorded for 30 s at 30 min. Young animals were assessed for 48 h and the last 24 h plotted, and middle aged and older animals were assessed for 72 h and the last 48 h plotted. Spontaneous locomotor activity (both horizontal and vertical) was concomitantly monitored with system-integrated infrared beams 0.5 in apart on the horizontal plane, providing a high-resolution grid covering the XY-planes, and the software provides counts of beam breaks by the mouse in 30-s epochs. Locomotor activity records all beam breaks and ambulatory activity records when the subject breaks consecutive beams showing movement along the sensors. This is relative to the subject wandering about the chamber. In the activity, VO_2_, and RER plots, the dark lines are the group averages, and the lighter lines are the SEM values at each timepoint.

## Quantitation of tryptophan metabolites

Kynurenines were measured in plasma from 20-month-old mice (males: QPRT^−/−^ *n* = 9, controls *n* = 10, and females: QPRT^−/−^ *n* = 10, controls *n* = 10). At the time of sacrifice, mice were anesthetized with isoflurane, and all tissues and organs were harvested and weighed by trained lab personnel. Whole blood was collected with BD Microtainer tube with Dipotassium EDTA (Becton, Dickinson and Company, Franklin Lakes, NJ) and spun at 1000 g at 4 °C for 13 min, then plasma was snap frozen in liquid nitrogen.

The target analytes L-tryptophan, nicotinamide and serotonin were obtained from Sigma (St Louis, MO), L-kynurenine, 3-hydroxykynurnenine, kynurenic acid and xanthurenic acid were obtained from MP Biomedicals (Santa Ana, CA) and 3-hydroxyanthranilic acid anthranilic acid were obtained from Fluka (Charlotte, NC). L-tryptophan 13C11, L-kynurenine sulfate:H2O (ring D4) and nicotinamide-D4 were obtained from Cambridge Isotopes (Tewksbury, MA), serotonin-D4, kynurenic acid-D5 and xanthurenic acid-D4 were obtained from Santa Cruz (Dallas, TX) and anthranilic acid-13C6 was obtained from ISOTEC (St Louis, MA) and xanthurenic acid-D4. All reagents used were of LC–MS purity and obtained from ThermoScientific unless otherwise stated. Bovine serum albumin (BSA) (Sigma) was prepared to a concentration of 70 g/L in LC–MS grade water. A stock calibration mix was prepared in BSA with tryptophan at 80,000 ng/mL, serotonin, quinolinic acid, nicotinamide and kynurenine at 8000 ng/mL, kynurenic acid, xanthurenic acid and anthranilic acid at 4000 ng/mL. A 9-point calibration curve was created with serial dilution covering 100–25,600 ng/mL for tryptophan, 2.5–2560 ng/mL for serotonin, quinolinic acid, nicotinamide and kynurenine, 5–1280 for kynurenic acid, xanthurenic acid and anthranilic acid and nicotinamide and 5.0–320 ng/mL for 3-hydroxyanthranilic acid and 3-hydroxykynurenine.

The internal standard solution was prepared with tryptophan-13C11, serotonin-D4 and kynurenine-D4 at 5 µg/mL, kynurenic acid-D5 at 0.5 µg/mL and nicotinamide-D4, anthranilic acid 13C6 and xanthurenic acid-D4 at 0.8 µg/mL in 90:10 water:methanol.

Fifty microliters of plasma and calibrator were aliquoted into microcentrifuge tubes. Ten microliters of the internal standard solution was added to each sample followed by vortex mixing; 400 µL of 8:1:1 acetonitrile:methanol:acetone was added to each sample followed by vortex mixing and then incubation at 4 °C for 30 min. The samples were then centrifuged at 20,000 rcf for 10 min at 4 °C; 400 µL of the supernatant was removed and transferred to a new microcentrifuge tube. The supernatant was dried in nitrogen gas at 30 °C. The residue was reconstituted in 50 µL of 0.1% formic acid in water. Blanks were prepared alongside.

Sample analysis was conducted on a ThermoScientific Q-Exactive Orbitrap with Dionex 3000 UHPLC. Briefly, samples were analyzed in positive heated electrospray ionization with the following source parameters: spray voltage = 3500 V, aux gas = 10, sheath gas = 50, capillary temperature = 325 °C, spray gas = 1 and S-lens RF level = 30. Separation was achieved on an ACE 18-PFP 100 × 2.1 mm, 2 µm column (Mac-Mod Analytical, Chadds Ford, PA) using a gradient with mobile phase A as 0.1% formic acid in water and mobile phase B as acetonitrile with a column temperature of 25 °C. Gradient elution was ramped from 0% B to 80% B over 13.0 min at 350 µL/min, and increased to 600 µL/min from 16.80 and 17.50 min for re-equilibration. The runtime was 20.50 min, and full scan at 35,000 mass resolution was acquired from 2 µL injection in positive ion mode. Calibration was conducted by extraction of the exact mass for each target analyte and reference to the respective internal standard.

## Complete blood count

Whole blood samples from 20 to 22-month-old male (QPRT^−/−^ *n* = 5, Control *n* = 7) and female (QPRT^−/−^ *n* = 5, Control *n* = 5) were analyzed for CBC by the Hemavet 950 (Drew Scientific, Waterbury, CT).

## Statistical analyses

Comparisons of individual metabolite values between QPRT −/− mice and control mice were carried out by two-sample, two-tailed *t* tests without assuming consistent SD, with statistical significance determined using the Holm-Bonferroni’s method for multiple comparisons correction with maximum family-wise error rate at 0.05. A *p* value less than 0.05 was considered significant. GTT data was analyzed using the two-way repeated measures analysis of variance (ANOVA). CBC data was analyzed by *t* test using False Discovery Rate method of significance with *Q* = 1%.

We used the general linear model (GLM) to analyze mass-dependent variables (energy expenditure, oxygen consumption, carbon dioxide production) from CLAMS data. Subsets of data categorized by time of day were analyzed. The data presented in the results tables represents the results of the statistical test for which the metabolic variable is being predicted by the group (and mass). The tables have *p* values from a model including the type of mass (total mass, lean mass or fat mass). If there was a significant interaction effect, it is displayed in the table; otherwise, it was removed from the model. When the interaction effect is removed, the test is analogous to an ANCOVA.

For the mass-independent metabolic variables (RER, Locomotor activity, ambulatory activity), the difference between groups is analyzed by a one-way ANOVA. Comparisons between young and old were analyzed by unpaired, two-tailed *t* test. Statistical analyses were performed using GraphPad Prism and CalR [36].

## Results

### QPRT^−/−^mice have altered levels of kynurenines and reduced nicotinamide

**Fig. 1:**
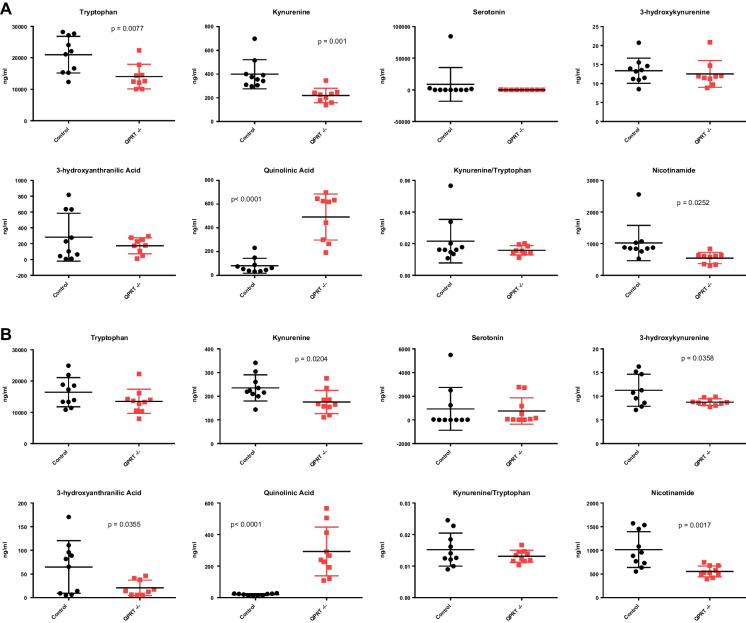
Quantitation of tryptophan metabolites. Plasma levels of tryptophan, kynurenine, serotonin, 3-hydroxykynurenine, 3-hydroxyanthranilic acid, quinolinic acid, the kynurenine/tryptophan ratio and nicotinamide determined by metabolomic approach in female (**A**) and male (**B**) QPRT^−/−^ mice (red) compared to controls (black), in ng/mL. Comparisons of individual metabolite values between QPRT −/− mice and control mice were carried out by two-sample, two-tailed *t* tests without assuming consistent SD, with statistical significance determined using the Holm-Bonferroni’s method for multiple comparisons correction

We measured circulating levels of tryptophan, serotonin and kynurenine pathway metabolites in 20-month-old QPRT^−/−^ mice, using LC–MS. Female QPRT^−/−^ mice had significantly reduced levels of tryptophan (*p* = 0.0077), kynurenine (*p* = 0.01) and nicotinamide (0.0252) and elevated levels of quinolinic acid (*p* < 0.0001) relative to controls (Fig. 1A). Male QPRT^−/−^ mice had reduced levels of kynurenine (*p* = 0.0204), 3-hydroxykynurenine (*p* = 0.0358), 3-hydroxyanthranilic acid (*p* = 0.0355) and nicotinamide (*p* = 0.0017), and elevated levels of quinolinic acid (*p* < 0.0001) relative to controls (Fig. 1B). While exhibiting some sexually dimorphic nuances, both sexes of QPRT^−/−^ mice had elevated quinolinic acid and reduced kynurenine and nicotinamide relative to controls.

## Metabolic changes in young QPRT^−/−^mice

**Fig. 2:**
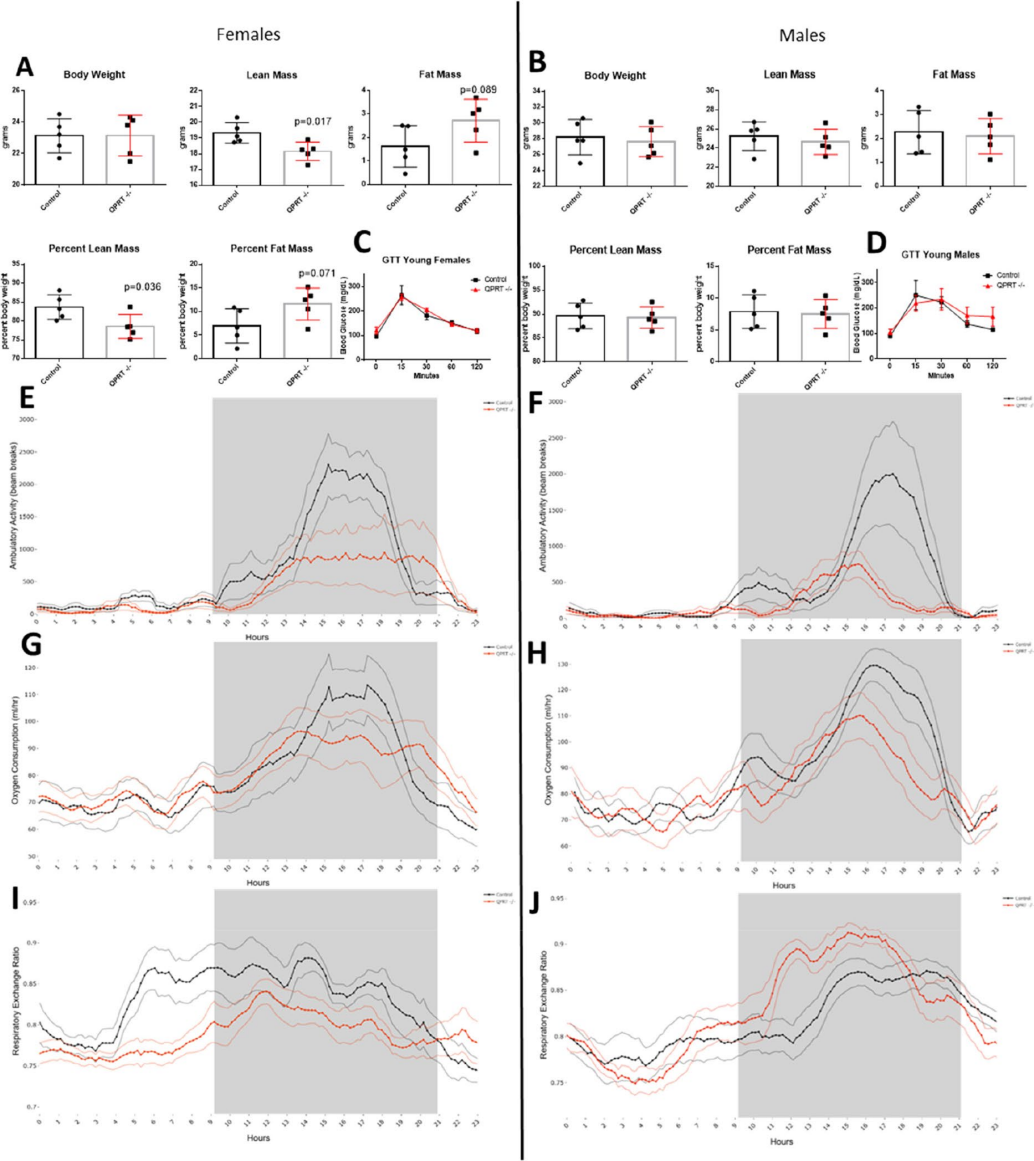
Body composition, energy metabolism and activity in young QPRT^−/−^ mice. Body weight, lean mass, and fat mass as well as lean mass and fat mass expressed as percentage of total body mass in female (**A**) and male (**B**) QPRT^−/−^ (red) and control (black) mice. Blood glucose levels (mg/dL) during glucose tolerance test (GTT) in female (**C**) and male (**D**) QPRT^−/−^ mice and controls. Ambulatory activity (number of beam breaks) (**E** and **F**), oxygen consumption (mL/h) (**G** and **H**), and respiratory exchange ratio (**I** and **J**) in QPRT^−/−^ (red) and control mice (black). Comparisons of body composition between QPRT −/− mice and control mice were carried out by two-sample, two-tailed *t* tests. GTT data was analyzed using the two-way repeated measures ANOVA. We used the general linear model (GLM) to analyze oxygen consumption and ambulatory activity and RER analyzed by a one-way ANOVA. In the activity, oxygen consumption and RER plots, the dark lines represent the group averages, and the lighter lines are the SEM values at each timepoint

At young age female QPRT^−/−^ mice had reduced lean mass (*p* = 0.017) and percent lean mass (*p* = 0.036) compared to controls (Fig. 2A). Young male QPRT^−/−^ mice had body composition similar to controls (Fig. 2B). Young female (Fig. 2C) and young male (Fig. 2D) QPRT^−/−^ mice responded to the glucose challenge similarly to controls. Both female (Fig. 2E) and male (Fig. 2F) QPRT^−/−^ mice had different activity patterns compared to controls during the dark portion of the study; however, these changes were not significant. Oxygen consumption (Fig. 2G, H) was not significantly altered in male or female QPRT^−/−^ mice; however, respiratory exchange ratio (RER) was significantly decreased during the light period only, in young female QPRT^−/−^ mice compared to controls (Fig. 2I). RER was not significantly altered in young male QPRT^−/−^ mice (Fig. 2J).

## Metabolic changes in middle-aged QPRT^−/−^mice

**Fig. 3:**
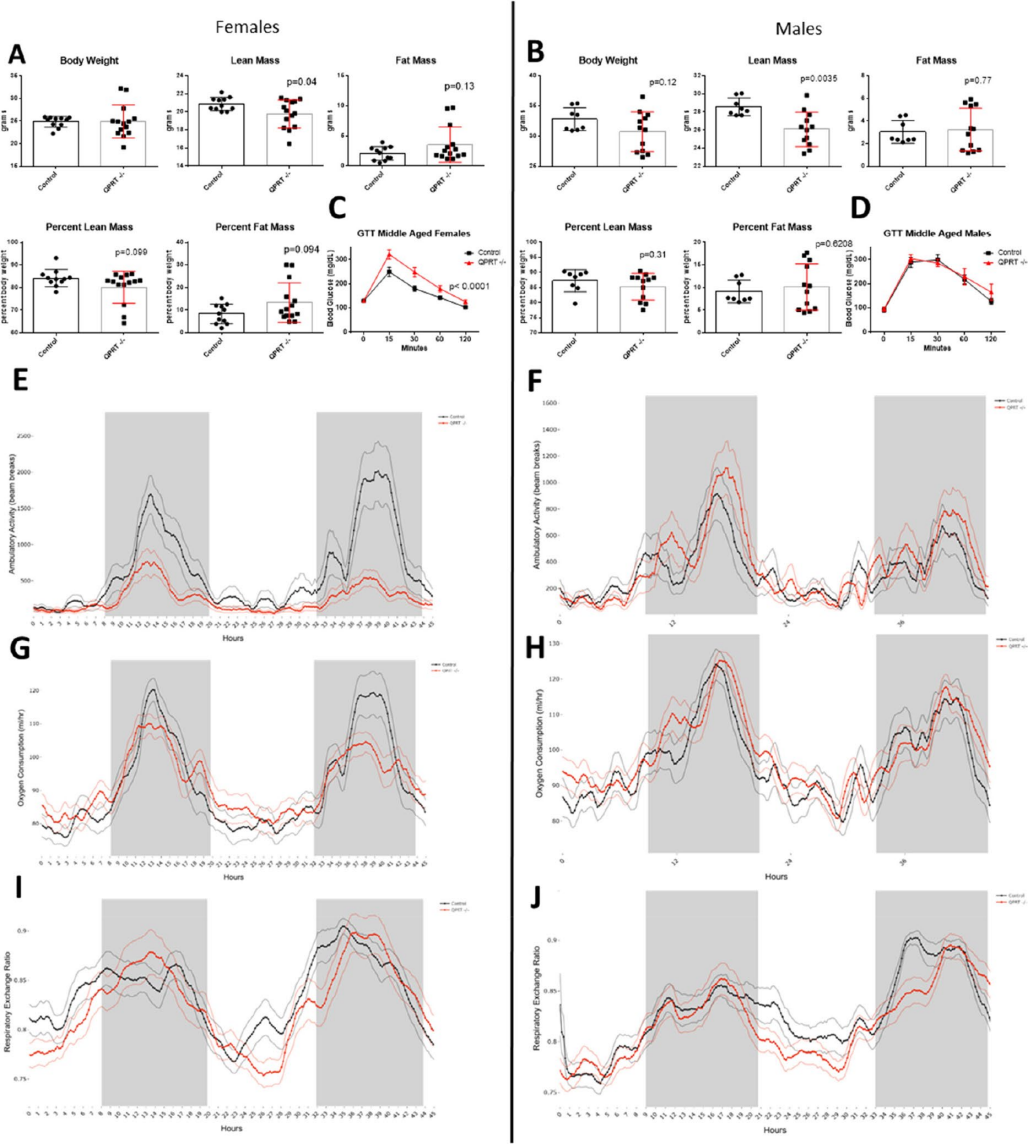
Body composition, energy metabolism and activity in middle aged QPRT^−/−^ mice. Body weight, lean mass, and fat mass as well as lean mass and fat mass expressed as percentage of total body mass in female (**A**) and male (**B**) QPRT^−/−^ (red) and control (black) mice. Blood glucose levels (mg/dL) during glucose tolerance test (GTT) in female (**C**) and male (**D**) QPRT^−/−^ mice and controls. Ambulatory activity (number of beam breaks) (**E** and **F**), oxygen consumption (mL/h) (**G** and **H**) and respiratory exchange ratio (**I** and **J**) in QPRT^−/−^ (red) and control mice (black). Comparisons of body composition between QPRT −/− mice and control mice were carried out by two-sample, two-tailed *t* tests. GTT data was analyzed using the two-way repeated measures ANOVA. We used the general linear model (GLM) to analyze oxygen consumption and ambulatory activity and RER analyzed by a one-way ANOVA. In the activity, oxygen consumption and RER plots, the dark lines represent the group averages, and the lighter lines are the SEM values at each timepoint

Middle-aged female and male QPRT^−/−^ mice had significantly reduced lean mass (*p* = 0.04 and *p* = 0.035, respectively) compared to controls (Fig. 3A, B). Middle-aged female QPRT^−/−^ mice had significantly reduced glucose clearance (*p* < 0.0001) (Fig. 3C), while males cleared glucose similarly to controls (Fig. 3D). Middle-aged female mice had significantly decreased ambulatory activity (*p* = 0.0053), notably during the dark period (*p* = 0.0029) when activity is at its peak (Fig. 3E, supplemental Table 3); locomotor activity was also significantly reduced during the entire recorded period (Full day: *p* = 0.0013, light period: *p* = 0.0264, dark period: *p* = 0.0021) (supplemental Table 3). Female mice had a significant mass effect when lean mass was the covariate for energy expenditure, oxygen consumption and carbon dioxide production (supplemental Table 3). In males there was a significant mass effect when total mass, lean mass and fat mass were used as the covariates for energy expenditure, oxygen consumption and carbon dioxide production at various time periods during the experiment (supplemental Table 4). Only during the dark period was there a significant group difference in VCO_2_ between QPRT^−/−^ mice and controls (supplemental Table 4) and a significant interaction in the relationship between VCO_2_ and lean mass driven by the increased rates of VCO_2_ relative to lean mass, in QPRT^−/−^ mice compared to controls (supplemental Table 4, supplemental Fig. 2). This was accompanied by significantly different relationship between lean mass and food consumption in QPRT^−/−^ mice (supplemental Fig. 3) with QPRT^−/−^ mice with having increased food consumption relative to their lean mass.

## Metabolic changes in old aged QPRT^−/−^mice

**Fig. 4:**
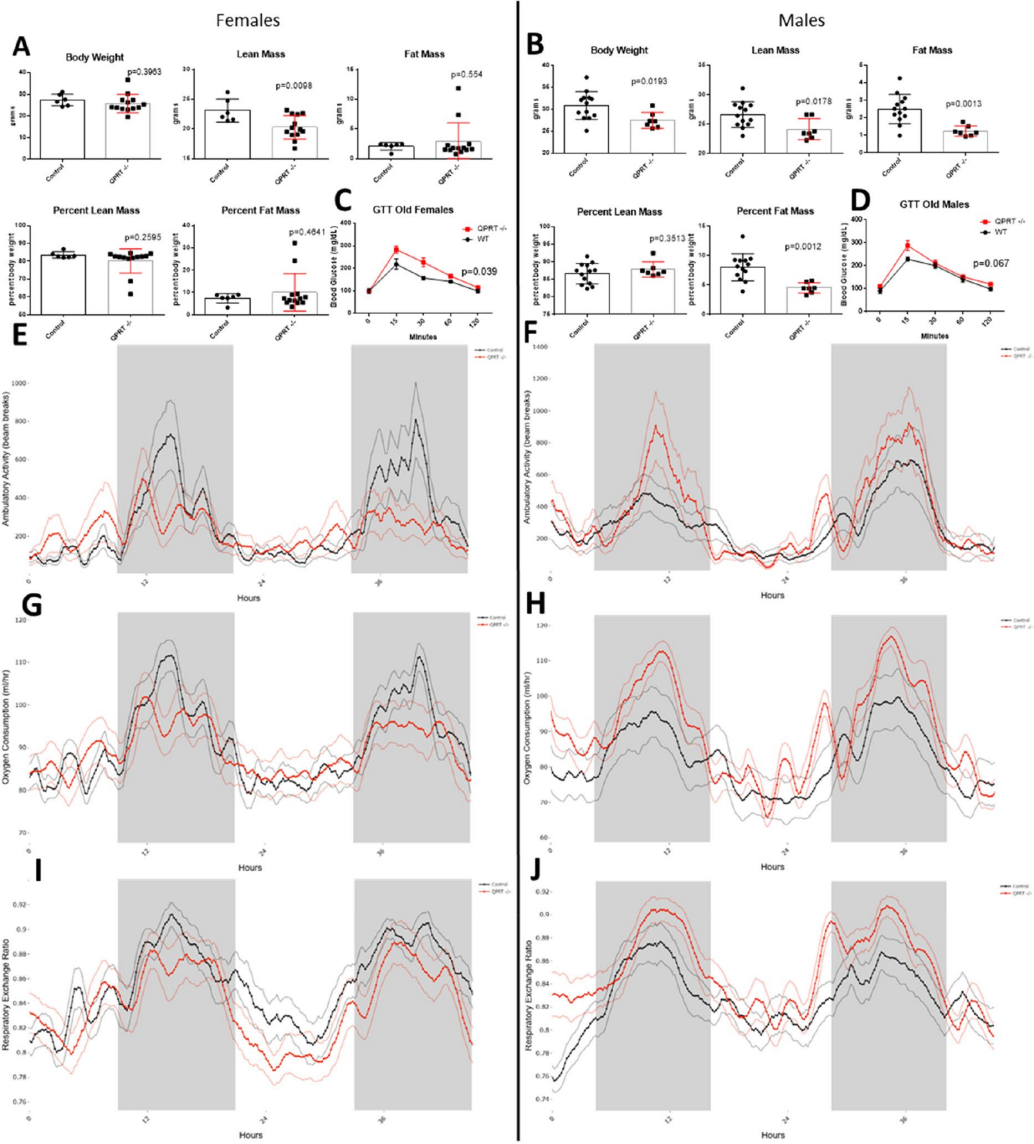
Body composition, energy metabolism and activity in old age QPRT^−/−^ mice. Body weight, lean mass, and fat mass as well as lean mass and fat mass expressed as percentage of total body mass in female (**A**) and male (**B**) QPRT^−/−^ (red) and control (black) mice. Blood glucose levels (mg/dL) during glucose tolerance test (GTT) in female (**C**) and male (**D**) QPRT^−/−^ mice and controls. Ambulatory activity (number of beam breaks) (**E** and **F**), oxygen consumption (mL/h) (**G** and **H**) and respiratory exchange ratio (**I** and **J**) in QPRT^−/−^ (red) and control mice (black). Comparisons of body composition between QPRT −/− mice and control mice were carried out by two-sample, two-tailed *t* tests. GTT data was analyzed using the two-way repeated measures ANOVA. We used the general linear model (GLM) to analyze oxygen consumption and ambulatory activity and RER analyzed by a one-way ANOVA. In the activity, oxygen consumption and RER plots, the dark lines represent the group averages, and the lighter lines are the SEM values at each timepoint

Older QPRT^−/−^ females had reduced lean mass compared to controls (*p* = 0.0098) (Fig. 4A), while older QPRT^−/−^ male mice had reduced body weight (*p* = 0.0193), reduced lean mass (*p* = 0.0178), reduced fat mass (*p* = 0.0013) and reduced percent fat mass (*p* = 0.0012) (Fig. 4B). GTT showed that female QPRT^−/−^ mice had significantly reduced glucose clearance compared to controls (*p* = 0.039) (Fig. 4C). QPRT^−/−^ male mice had a trend for decreased clearance; however, this did not reach significance (Fig. 4D). Male and female QPRT^−/−^ mice had different patterns of ambulatory activity, oxygen consumption and RER with female QPRT^−/−^ mice having blunted levels during peak activity (Fig. 4E, G, I), while male QPRT^−/−^ mice had trends for increased levels during peak activity (Fig. 4H, J); however, none of these changes reached statistical significance (supplemental tables 5 and 6). Carbon dioxide production was significantly increased relative to lean mass in QPRT^−/−^ mice compared to controls (supplemental Table 6, supplemental Fig. 4).

**Fig. 5:**
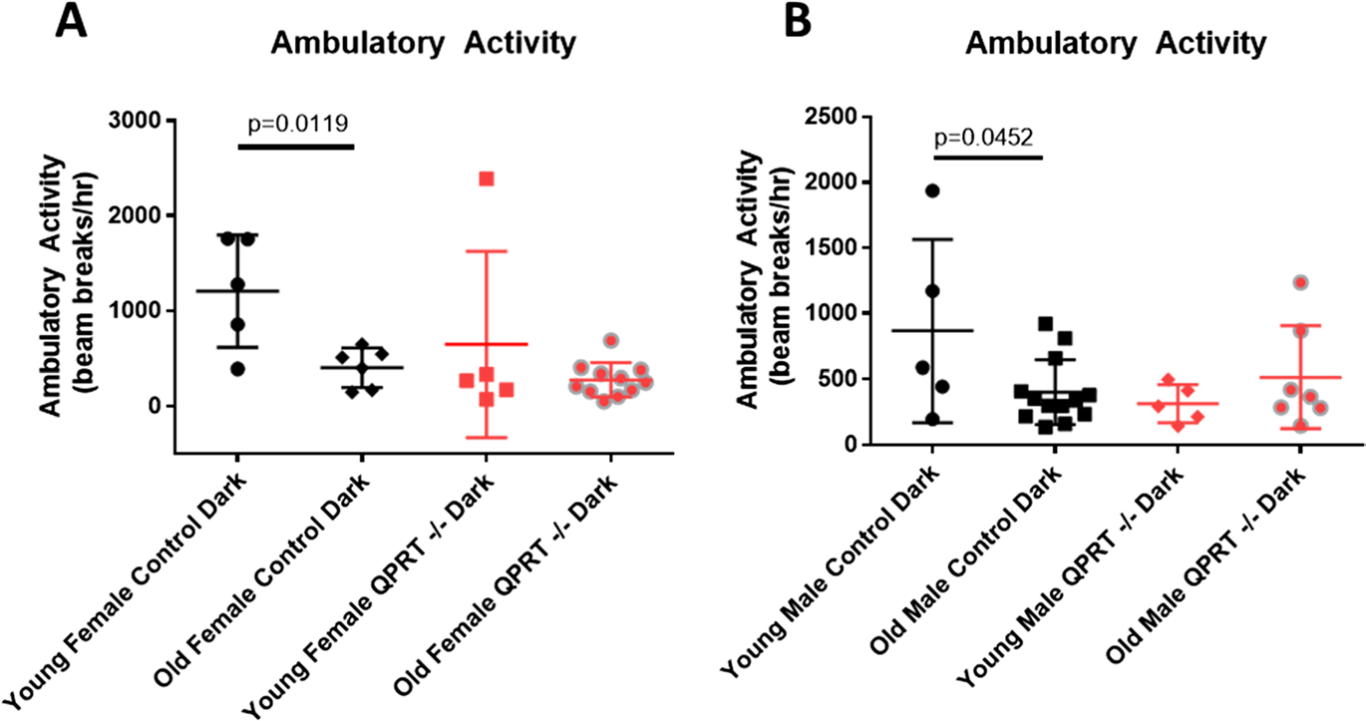
Comparison in rates of ambulatory activity in young and old mice. We compared levels of average ambulatory activity (beam breaks/h) between young and old, female (**A**) and male (**B**), QPRT^−/−^ and control mice. Comparisons between young and old were analyzed by unpaired, two-tailed *t* test

When we compared rates of dark period ambulatory activity between old and young mice, we saw that both female (*p* = 0.0119) and male (*p* = 0.0452) control mice had significant declines in activity with age. Interestingly, QPRT^−/−^ mice had reduced activity at young age which persisted into old age (Fig. 5A, B).

Lastly, we compared complete blood count measures in old-aged female and male QPRT^−/−^ and control mice (supplemental Table 7). Interestingly, while male QPRT^−/−^ mice were similar to their controls, female QPRT^−/−^ mice had decreased red blood cells (*p* = 0.001), hemoglobin (*p* = 0.002), hematocrit (*p* = 0.003), mean corpuscular hemoglobin (*p* = 0.001), immature reticulocyte fraction (*p* = 0.001) and reticulocyte low, medium and high fluorescence rate (*p* = 0.001).

## Discussion

This study has shown that QPRT^−/−^ mice have reduced circulating levels of tryptophan and other upstream kynurenines, a reduction in physical activity at young age which is accompanied by reduced total lean mass and in females, reduced percent lean mass and reduced glucose clearance during GTT. To our knowledge, this is the first study to measure the levels of circulating kynurenines in aged QPRT^−/−^ mice, and the first to examine glucose handling and energy metabolism, across the life course of QPRT^−/−^ mice. There were several notable findings from these experiments.

The first surprising results from this study include the decreased concentrations of circulating kynurenine-related metabolites in QPRT^−/−^ mice. We saw reduced tryptophan and kynurenine in 20-month-old female QPRT^−/−^ mice and reduced kynurenine, 3-hydroxykynurenine and 3-hydroxyanthranilic acid in male QPRT^−/−^ mice. We expectedly saw elevated levels of quinolinic acid in both sexes; however, this was accompanied by significantly reduced levels of nicotinamide. This indicates that de novo biosynthesis through the KP is a significant source of circulating nicotinamide in mice even though the diet is supplemented with nicotinic acid (115 mg/kg chow). Reduced circulating levels of tryptophan are known to occur with aging and inflammation in numerous species including mice and humans; however, lowered tryptophan is typically accompanied by elevated levels of kynurenine [10]. Here, tryptophan, kynurenine and in males, 3-hydroxykynurenine and 3-hydroxyanthranilic are decreased in aged QPRT^−/−^ mice. One possible explanation is that NADPH and other nicotinyl derivatives such as, NADH, nicotinamide mononucleotide, nicotinamide and nicotinic acid are allosteric inhibitors of TDO [37, 38]. Reduced levels of nicotinamide (and presumably other nicotinyl derivatives) in QPRT −/− mice result in removal of the negative feedback inhibition. Without this inhibition, increased tryptophan catabolism occurs through the kynurenine pathway, thus depleting precursors. This also indicates that systemic elevations in quinolinic acid are not inhibitory to the KP even at levels tenfold greater than that of controls. Enzyme levels do not explain the lowered levels of kynurenines as previous studies have shown that 16-month-old QPRT^−/−^ mice have similar levels of kynurenine pathway enzymes as controls in tissues where they were measured, including IDO, TDO, KMO, kynurenine aminotransferase (KAT II), kynureninase (KYNase), 3-hydroxyanthranilic acid 3,4-dioxygenase (3-HAO) and 2-amino-3-carboxymuconate-6-semialdehyde (ACMSD) [39].

The liver makes NAD + de novo from tryptophan, releasing nicotinamide into the blood, which supports NAD + biosynthesis in the rest of the body [13]. Nicotinamide is the major circulating NAD + precursor in mice, so decreased levels seen in QPRT −/− mice likely contribute to metabolic dysfunction and functional decline seen in this study and in previous studies [35]. Numerous studies have shown that there is an age-dependent decline in NAD^+^ levels, which ranges from 10 to 65%, depending on the organ and age [40]. Because NAD + is central to cellular respiration as well as influences a number of cellular processes, is it is likely that *Qprt* gene disruption affects aging-related phenotypes by decreasing the available nicotinamide (and other nicotinyl derivatives) pool by roughly half, as seen in this study.

Another factor that may contribute to altered metabolism and functional decline are the elevated levels of quinolinic acid. Previous studies from our lab and others have shown that elevated QA can confer neurotoxicity via the N-methyl-D-aspartate (NMDA) receptor, to nerves including spinal motor neurons of QPRT^−/−^ mice indicating that chronic exposure to elevated levels of QA can lead to declines in neuromuscular function [35]. This previous study also showed that declines in QPRT^−/−^ mice begin in early and persist into old age. The results of ambulatory activity measurements are in agreement with this progression. While both male and female controls had significant decreases in peak activity (dark period) with age, young QPRT^−/−^ mice had activity levels that were not different than older QPRT^−/−^ mice. Young male and female QPRT^−/−^ mice already had numerically reduced activity which persisted into old age. It is likely that reduced activity influences glucose clearance in QPRT^−/−^ middle-aged females since reduced activity is associated with decrease glucose clearance [41] while elevations in quinolinic acid are known to inhibit gluconeogenesis [42].

Another similarity between this study and our previous studies are the findings on lean mass. We previously showed that QPRT^−/−^ mice had significantly smaller tibialis anterior than wild-type mice at young and middle age, in both males and females, suggesting that lean body mass is reduced in QPRT^−/−^ mice. This study confirms that QPRT^−/−^ mice had decreased total lean mass at several points throughout the life course, but only young female QPRT^−/−^ mice had decreased percent lean mass, relative to controls. NMR-based body composition counts all non-fat and non-bone tissue in the mouse as lean mass; therefore, decreases in small muscles may be imperceivable relative to total lean mass.

There was a significant mass effect on energy expenditure, oxygen consumption and carbon dioxide production when lean mass, and in middle-aged males, total mass, lean mass and fat mass were used as the covariate, at various timepoints during the experiment in middle-aged male and female mice. Interestingly, there was a significant effect of lean mass, on carbon dioxide production in middle-aged males during the dark period. Further, there was a group difference and a significant interaction in the relationship between VCO_2_ and lean mass during the dark period in middle-aged QPRT^−/−^ males compared to controls. This difference was accompanied by increased food consumption and numerically elevated activity in middle-aged QPRT^−/−^ male mice. This elevation is interesting considering these animals were lean and had reduced percent fat mass. Beginning at middle age, QPRT^−/−^ mice had sexually dimorphic metabolic characteristics with females having reduced activity and males having numerically increased activity, increased food consumption and increased VCO2. The numerous differences in CBC data in females but not male QPRT^−/−^ mice add to the number of sexually dimorphic phenotypic characteristics of this mouse. The mechanisms of sexually dimorphic response to *Qprt* gene disruption warrants further investigation in future studies.

Limitations of this study include that it is cross-sectional and not longitudinal and conducted on mice of one genetic background. This study was designed to identify changes in metabolism, and mechanisms of altered metabolism were not studied here. Levels of tryptophan derivatives were only measured in one age group, and nicotinyl species that we were able to measure was limited to nicotinamide. The behavioral implications of elevated QA were not investigated here. Studies investigating the response of QPRT^−/−^ mice to exercise or stressors such as temperature stress and high-fat diet must be left for future studies.

Supplementary information.

## Supplementary Information

Below is the link to the electronic supplementary material.

Supplementary file1 (DOCX 1357 KB)

## Data Availability

Authors are prepared to send relevant documentation or data in order to verify the validity of the results presented.
