# Supplementary material for: Kynurenine pathway dysregulation via loss of QPRT drives declines in activity and altered metabolism in mice
Source: GeroScience. 2025 Jul 10;48(2):2281–94. doi: 10.1007/s11357-025-01735-1 (PMC12972454; doi:10.1007/s11357-025-01735-1)
Supplement: Supplementary file 1 — Supplementary file1 (DOCX 1357 KB) [file 11357_2025_1735_MOESM1_ESM.docx]

# Supplemental Fig 1A Female Kynurenines

**Kynurenic Acid Xanthurenic Acid Anthranilic Acid**

**W**

**T**

**Q**

**P**

**R**

**T**

**K**

**O**

**0**

**20**

**4**

**0**

**6**

**0**

**n**

**g**

**/**

**m**

**l**

**W**

**T**

**Q**

**P**

**R**

**T**

**K**

**O**

**-5**

**0**

**5**

**1**

**0**

**15**

**2**

**0**

**n**

**g**

**/**

**m**

**l**

**W**

**T**

**Q**

**P**

**R**

**T**

**K**

**O**

**0**

**20**

**40**

**60**

**80**

**100**

**n**

**g**

**/**

**m**

**l**

**Supplemental Figure 1A. Quantitation of tryptophan metabolites**. Plasma levels of kynurenic acid, xanthurenic acid and anthranilic acid determined by metabolomic approach in female QPRT -/- mice (red) compared to controls in ng/ml. Comparisons of individual metabolite values between QPRT-/- mice and control mice were carried out by 2-sample, 2 tailed t tests without assuming consistent SD, with statistical significance determined using the Holm-Bonferroni’s method for multiple comparisons correction.

# Supplemental Fig 1B Male Kynurenines

**Kynurenic Acid Xanthurenic Acid Anthranilic Acid**

**W**

**T**

**Q**

**P**

**R**

**T**

**K**

**O**

**0**

**5**

**10**

**15**

**n**

**g**

**/**

**m**

**l**

**W**

**T**

**Q**

**P**

**R**

**T**

**K**

**O**

**-2**

**0**

**2**

**4**

**6**

**8**

**n**

**g**

**/**

**m**

**l**

**W**

**T**

**Q**

**P**

**R**

**T**

**K**

**O**

**0**

**10**

**20**

**30**

**40**

**n**

**g**

**/**

**m**

**l**

**Supplemental Figure 1B. Quantitation of tryptophan metabolites**. Plasma levels of kynurenic acid, xanthurenic acid and anthranilic acid determined by metabolomic approach in male QPRT -/- mice (red) compared to controls in ng/ml. Comparisons of individual metabolite values between QPRT-/- mice and control mice were carried out by 2-sample, 2 tailed t tests without assuming consistent SD, with statistical significance determined using the Holm-Bonferroni’s method for multiple comparisons correction.

# Supplemental Fig 2


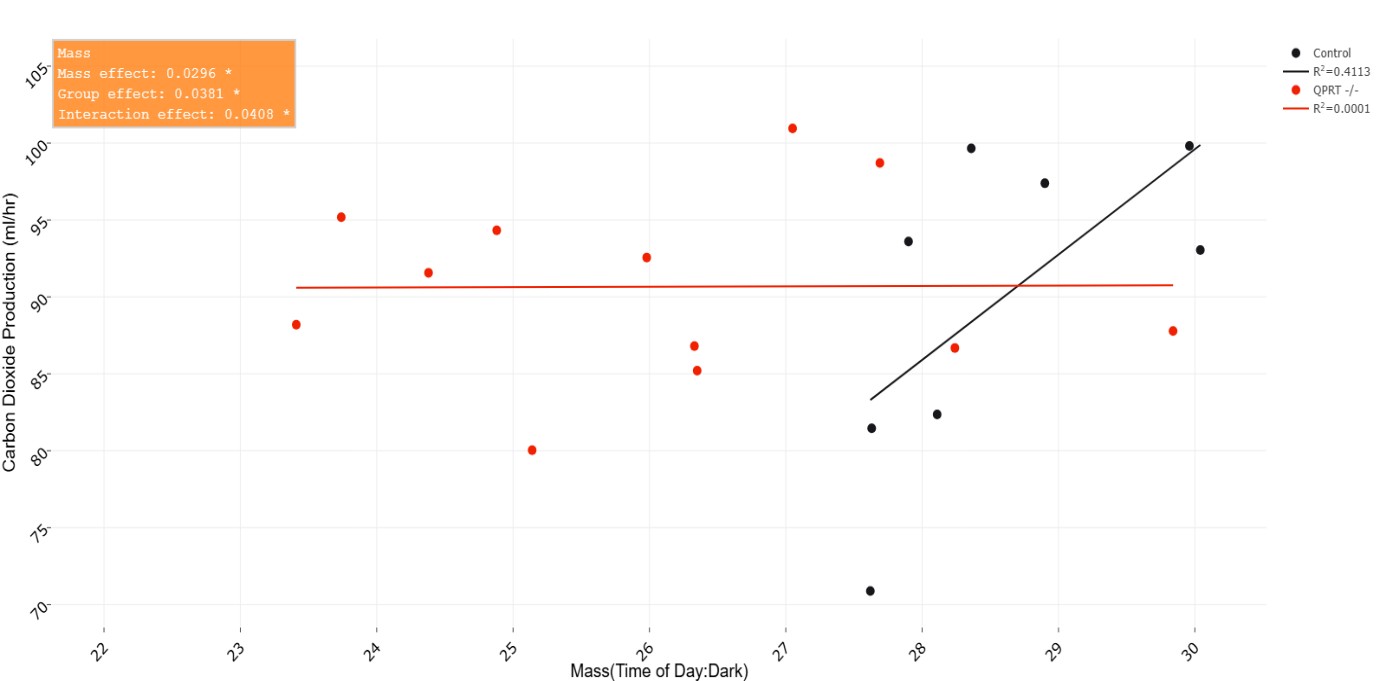


**Supplemental Figure 2**. Linear regression-based analysis of carbon dioxide production and lean mass in middle aged male QPRT -/- and control mice.

#
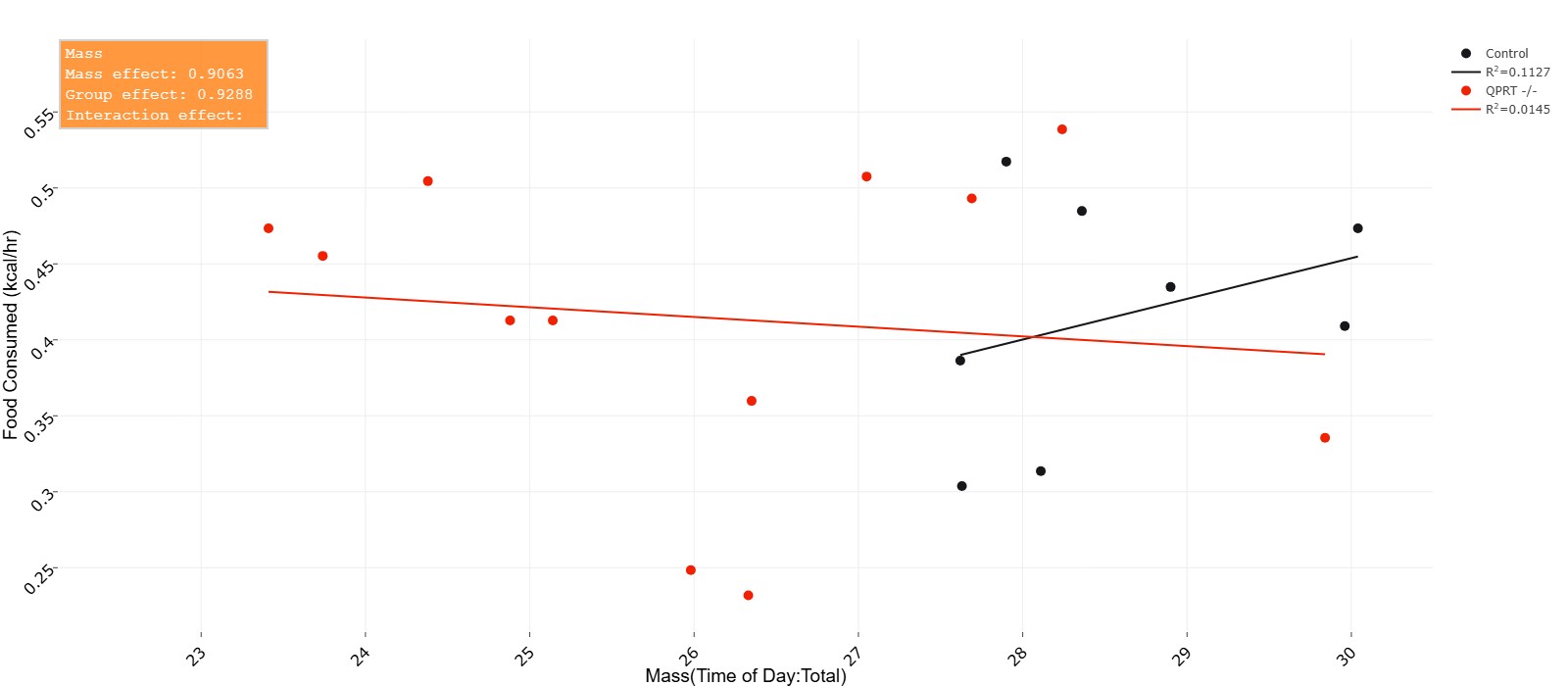
Supplemental Fig 3

| **Middle aged Male GLM** | | | |
| --- | --- | --- | --- |
|  | Dark | | |
| Mass Effect: Lean Mass | Mass | Group | Interaction |
| Food consumption (kcal/period) | 0.0579 | **0.0328** | **0.032** |

**Supplemental Figure 3**. Linear regression-based analysis of food consumption and lean mass in middle aged male QPRT -/- and control mice.

# Supplemental Fig 4 Old Age Males


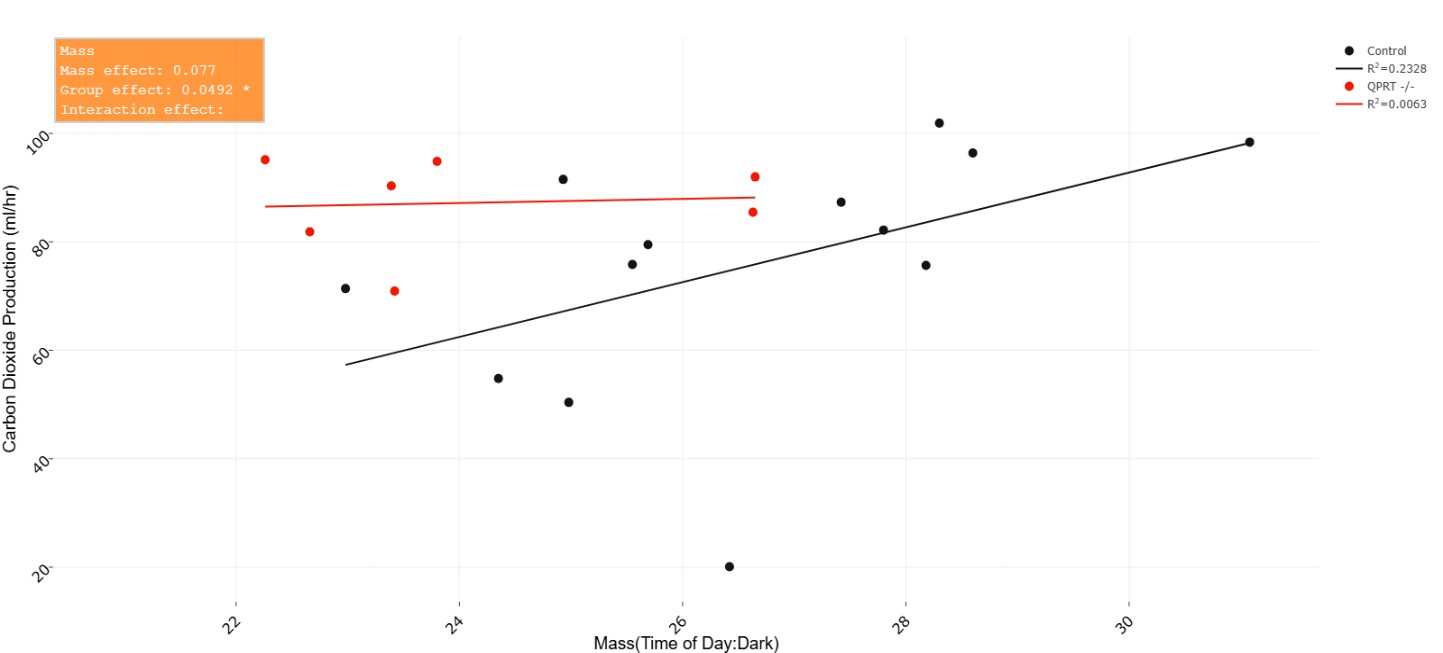


**Supplemental Figure 4**. Linear regression-based analysis of carbon dioxide production and lean mass in old aged male QPRT -/- and control mice.

##
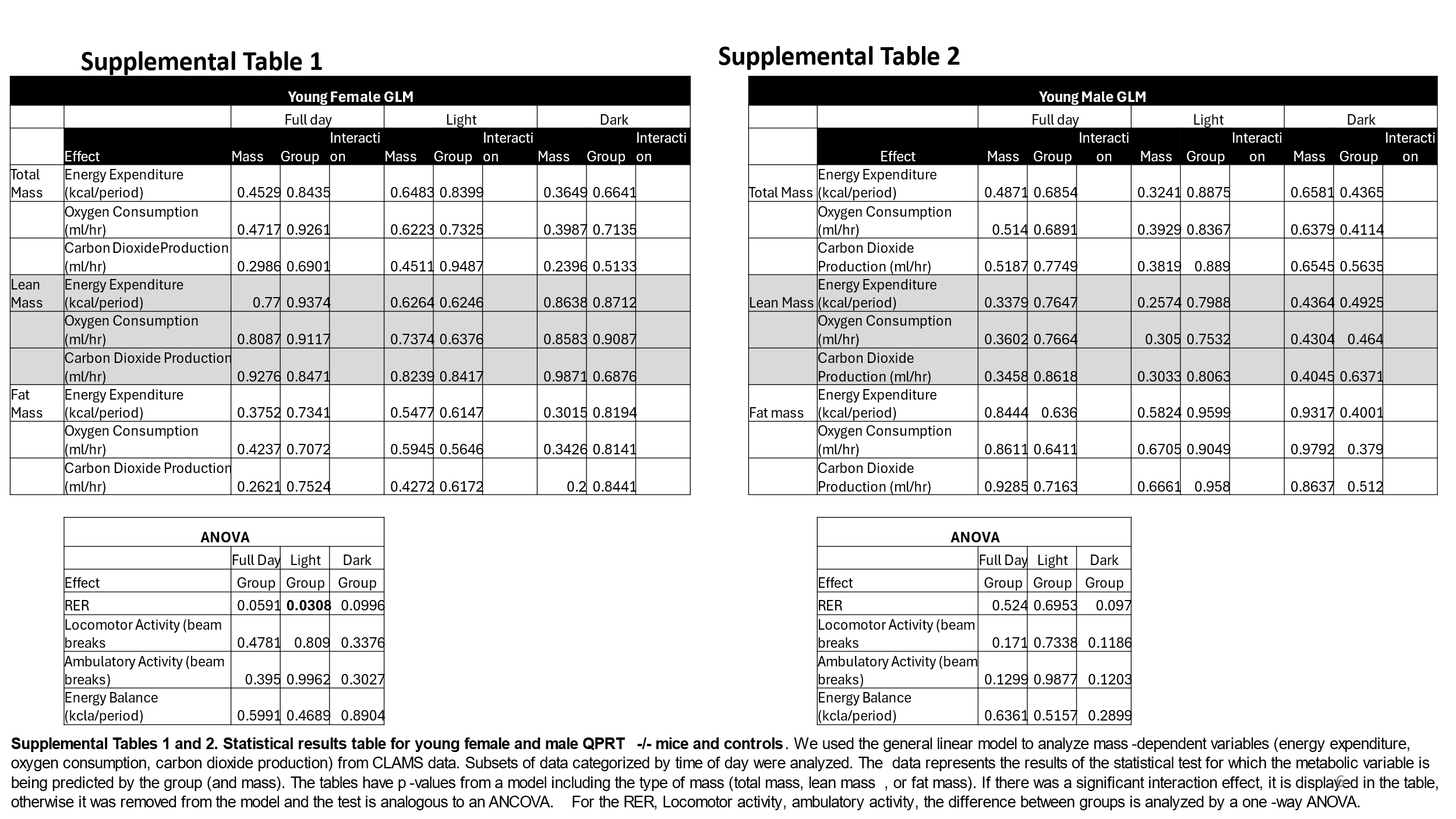


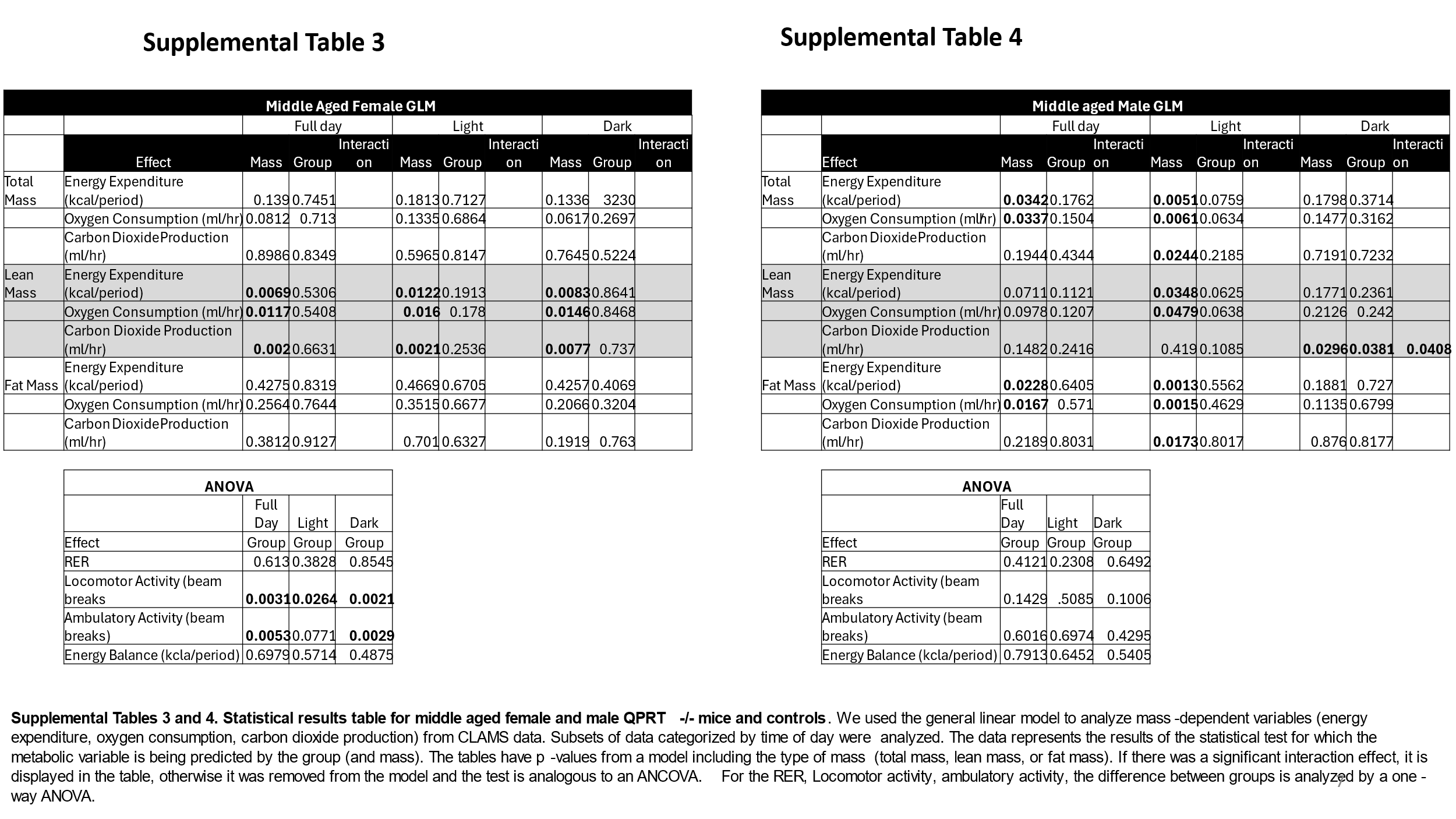


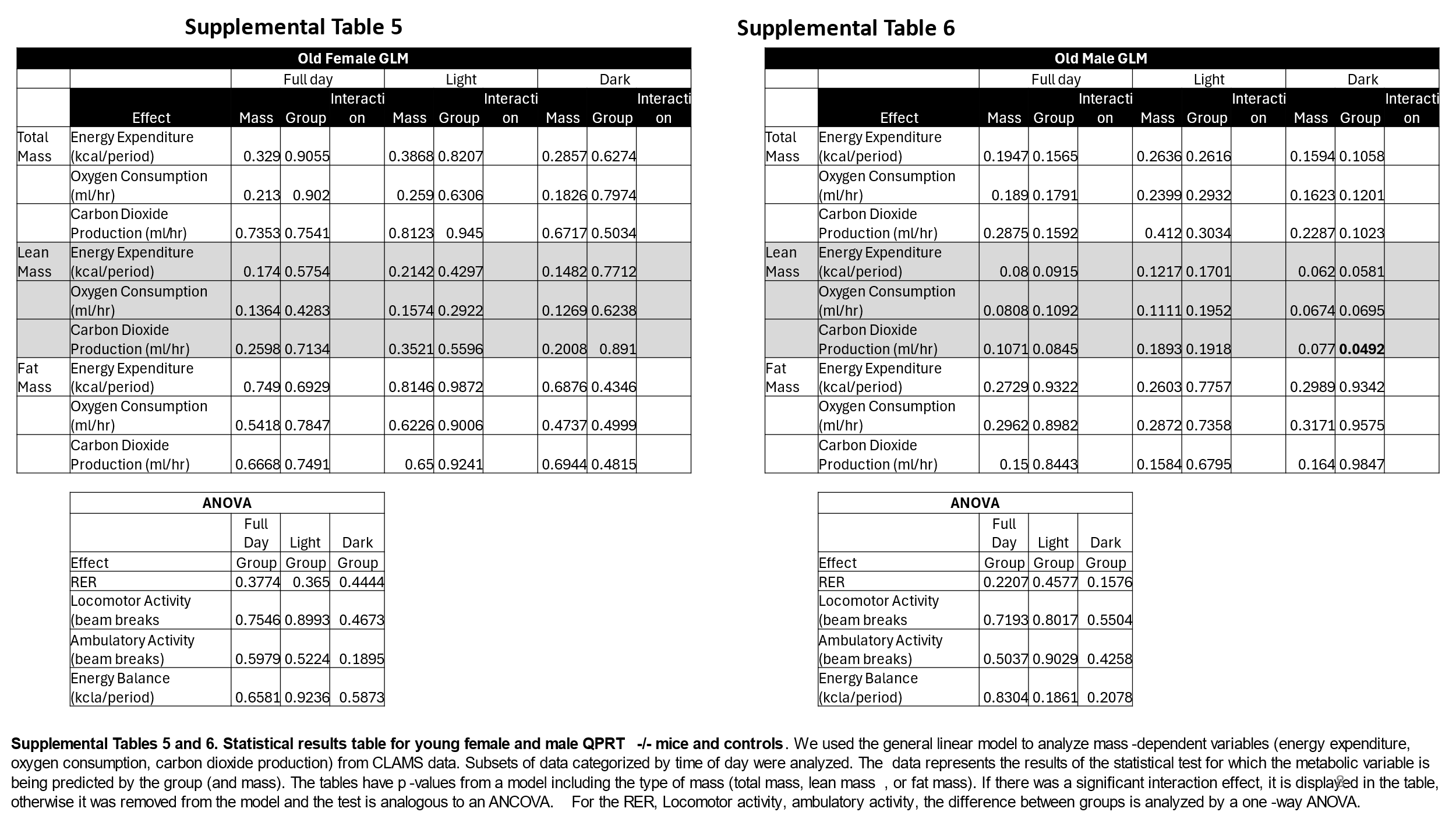


# Supplemental Table 7

|  |  | Male |  |  | Female |  |
| --- | --- | --- | --- | --- | --- | --- |
| **CBC Parameter** | **Control SEM** | **QPRT -/- SEM** | **p-value** | **Control SEM** | **QPRT -/- SEM** | **p-value** |
| Red blood cell [M/uL] | 6.96 1.19 | 5.88 0.96 | 0.433 | **9.33 0.10** | **5.56 0.67** | **0.001*** |
| Hemoglobin [g/dL] | 11.25 1.81 | 9.54 1.28 | 0.363 | **14.40 0.14** | **9.24 1.13** | **0.002*** |
| Hematocrit [%] | 37.13 5.91 | 31.64 3.49 | 0.318 | **46.72 0.53** | **32.32 3.28** | **0.003*** |
| Mean cell volume [fl] | 55.55 3.53 | 57.14 5.43 | 0.807 | 50.10 0.45 | 59.54 4.78 | 0.085 |
| Mean corpuscular hemoglobin [pg] | 16.60 0.62 | 16.76 0.88 | 0.889 | **15.44 0.10** | **16.60 0.21** | **0.001*** |
| Mean corpuscular hemoglobin concentration [g/dl] | 30.07 1.62 | 29.74 1.03 | 0.775 | 30.84 0.14 | 28.46 1.88 | 0.242 |
| Red blood cell distribution width (SD) [fl] | 35.85 1.80 | 37.24 5.72 | 0.806 | 31.90 0.71 | 42.22 6.72 | 0.165 |
| Red blood cell distribution width (CV) [%] | 21.63 0.81 | 20.80 0.90 | 0.535 | 23.26 0.29 | 21.54 1.25 | 0.215 |
| Reticulocytes [K/ul] | 308.75 58.62 | 314.54 102.37 | 0.963 | 229.60 21.47 | 634.44 235.86 | 0.126 |
| Immature reticulocyte fraction [%] | 44.57 7.62 | 51.52 4.75 | 0.379 | **36.38 3.05** | **61.74 4.07** | **0.001*** |
| Reticulocyte low flourescence rate [%] | 55.43 7.62 | 48.48 4.75 | 0.379 | **63.62 3.05** | **38.26 4.07** | **0.001*** |
| Reticulocyte medium flourescence rate [%] | 26.33 3.85 | 22.40 1.48 | 0.099 | **25.98 0.97** | **17.56 1.41** | **0.001*** |
| Reticulocyte high flourescence rate [%] | 18.23 6.04 | 29.12 6.09 | 0.263 | **10.40 2.88** | **44.18 5.28** | **0.001*** |
| Reticulocyte hemoglobin [pg] | 17.62 0.57 | 17.62 0.53 | 0.997 | 16.78 0.12 | 17.08 0.32 | 0.398 |
| Platelet [K/ul] | 708.17 228.37 | 613.40 177.99 | 0.769 | **849.40 108.54** | **300.40 130.81** | **0.012** |
| Platelet distribution width [fL] | 6.35 0.08 | 7.82 1.40 | 0.275 | 6.34 0.08 | 7.83 1.07 | 0.108 |
| Mean platelet volume [fL] | 7.57 0.37 | 8.70 1.03 | 0.258 | **7.10 0.10** | **10.88 1.37** | **0.025** |
| Platelet large cell ratio [%] | 3.23 0.38 | 7.40 4.65 | 0.349 | **1.88 0.09** | **8.23 2.84** | **0.022** |
| Plateletcrit [%] | 0.54 0.17 | 0.47 0.12 | 0.782 | **0.60 0.07** | **0.27 0.09** | **0.022** |
| White blood cell [K/ul] | 1.47 0.37 | 1.04 0.21 | 0.359 | 2.90 0.67 | 2.06 0.68 | 0.411 |
| Neutrophils [K/ul] | 0.46 0.10 | 0.43 0.07 | 0.822 | 0.69 0.13 | 0.82 0.33 | 0.708 |
| Lymphocytes [K/ul] | 0.85 0.22 | 0.51 0.13 | 0.241 | 2.03 0.51 | 0.88 0.26 | 0.108 |
| Monocytes [K/ul] | 0.09 0.03 | 0.07 0.02 | 0.714 | 0.10 0.02 | 0.32 0.15 | 0.150 |
| Eosinophils [K/ul] | 0.08 0.03 | 0.03 0.01 | 0.252 | 0.08 0.03 | 0.03 0.01 | 0.145 |
| Reticulocytes % | 6.44 7.87 | 8.42 5.21 | 0.748 | 2.47 0.24 | 14.24 7.55 | 0.158 |
| Neutrophils % | 34.10 5.56 | 41.60 1.86 | 0.086 | 24.64 1.20 | 36.60 9.41 | 0.196 |
| Lymphocytes % | 55.68 8.17 | 47.48 3.45 | 0.068 | 69.00 1.98 | 47.90 10.87 | 0.069 |
| Monocytes % | 5.72 1.45 | 6.68 1.48 | 0.652 | **3.86 0.88** | **13.60 2.97** | **0.010** |
| Eosinophils % | 4.22 1.20 | 3.70 1.11 | 0.767 | 2.50 0.62 | 1.23 0.21 | 0.122 |

**Supplemental Table 7. CBC from old aged male and female QPRT -/- mice and controls.** Comparisons of CBC parameters between QPRT-/- mice and control mice were carried out by 2-sample, 2 tailed t tests without assuming consistent SD, with statistical significance determined using False discovery rate method of significance determination. Values in bold font have p-values <0.05 and * indicates discoveries determined by FDR test.
